# Higher Essential Amino Acid and Crude Protein Contents in Pollen Accelerate the Oviposition and Colony Foundation of *Bombus breviceps* (Hymenoptera: Apidae)

**DOI:** 10.3390/insects14020203

**Published:** 2023-02-17

**Authors:** Chang-Shi Ren, Zhi-Min Chang, Lei Han, Xiang-Sheng Chen, Jian-Kun Long

**Affiliations:** 1 Key Laboratory of Animal Genetics, Breeding and Reproduction in the Plateau Mountainous Region, Ministry of Education/College of Animal Science, Guizhou University, Guiyang 550025, China; 2 Institute of Entomology/Special Key Laboratory for Developing and Utilizing of Insect Resources, Guizhou University, Guiyang 550025, China

**Keywords:** bumblebees, pollen nutrition, amino acid, crude protein, oviposition, Apidae

## Abstract

**Simple Summary:**

*Bombus breviceps* is an important native species of bumblebee in southern China. As a pollinator, it has the potential for domestication and commercial rearing. In this study, in order to identify the nutritional requirements for the egg-laying, hatching, and colony foundation stages, we selected three common local types of pollen, including camellia pollen, oilseed rape pollen, and apricot pollen, as well as mixtures of them, to feed *B. breviceps* queens to evaluate the reproductive performance, and feasibility of these pollens as a diet and the nutritional requirements. The results showed that the pollen with a higher essential amino acid content had advantages in laying time, the number of eggs laid, larval ejection, and the time of first worker emergence, while the pollen with a higher crude protein content had advantages in the time the colony took to reach ten workers. These results may guide the selection of feed in *B. breviceps* artificial feeding and help to explore the nutritional requirements in oviposition and the colony stage from the perspective of conventional pollen.

**Abstract:**

Pollen is an important source of nutrition for bumblebees to survive, reproduce, and raise their offspring. To explore the nutritional requirements for the egg laying and hatching of queenright *Bombus breviceps* colonies, camellia pollen, oilseed rape pollen, apricot pollen, and mixtures of two or three types of pollen in equal proportions were used to feed the queens in this study. The results showed that the camellia pollen with a higher essential amino acid content was superior to the pollen with a lower essential amino acid content in the initial egg-laying time (*p* < 0.05), egg number (*p* < 0.05), larval ejection (*p* < 0.01), time of first worker emergence (*p* < 0.05), and the average weight of workers in the first batch (*p* < 0.01). It took less time for colonies under the camellia pollen and camellia–oilseed rape–apricot pollen mix treatments, both with a higher crude protein content, to reach ten workers in the colony (*p* < 0.01). On the contrary, the queens fed apricot pollen never laid an egg, and larvae fed oilseed rape pollen were all ejected—both pollens with a lower essential amino acid content. The results emphasize that the diet should be rationally allocated to meet the nutritional needs of local bumblebees at various stages when guiding them to lay eggs, hatch, and develop a colony.

## 1. Introduction

Bumblebees are important pollinators of many wild plants and crops, and they play vital roles in natural and agricultural ecosystems [1,2]. In recent years, declines in the populations and species of wild bumblebees have posed a threat to crop pollination and biodiversity [3]. *Bombus terrestris*, as the most successful commercial bumblebee in Europe, has been widely introduced into many countries for pollination [1,4]. However, *B. terrestris*, as an alien species, can escape or be deliberately released during pollination, potentially spreading new diseases and parasites [5,6] and competing with native bumblebees for food and nesting sites [7,8], which may disrupt the survival and reproduction of the other bumblebee species in the local area [9,10,11]. Biological invasions of native bee species, vegetation, and ecosystems, have possibly been caused by the alien species *B. terrestris* naturalized [7,12,13,14], with adverse effects on local ecosystems. Given this situation, many countries are trying to cultivate native bumblebee species, such as *Bombus impatiens* and *Bombus occidentalis* in North America [15], *Bombus ignitus* in South Korea [12,16], *Bombus hypocrita* and *Bombus ignitus* in Japan [17,18,19], and *Bombus ignitus, Bombus lucorum, Bombus pyrosoma, Bombus disciplines, Bombus lantschoolensis* and *Bombus patagiatus* in northern China [20,21]. However, the above six native Chinese bumblebees still have more deficiencies in the colony foundation rates, the number of workers, and the success rate of queen mating, compared with *B. terrestris* [22], have not been used commercially yet. *Bombus breviceps*, as a native Chinese species mainly distributed in southern China [23,24], is an important pollinator for many plants, such as Leguminosae, Solanaceae, Buddlejaceae, Clusiaceae, Cucurbitaceae, Lamiaceae, and Nyctaginaceae [21,25,26]. Recent research shows that *B. breviceps*, having higher colony foundation rates, a large number of workers, and new virgin queens, is a promising candidate for domestication and commercial rearing [27].

The diet and nutritional requirements are key factors for the artificial feeding of bumblebees. In wild nature, bumblebees obtain their macronutrients including carbohydrates, proteins, and lipids from floral nectar and pollen, to meet their nutritional demands [28]. Pollen is an important food to promote the development of their ovaries [29]. It provides proteins, lipids, carbohydrates, and micronutrients needed for the growth and development of larvae, pupae, and colonies [30]. The nutritional value of pollen, especially in the amount and proportion of nutrients, is a key factor in feeding: for example, in *B. terrestris* microcolonies, salix and camellia pollens with a higher amino acid content led to a higher number of eggs laid, and living larvae, and a lower rate of larva ejection, respectively [31,32]. Further, the number of drones in a microcolony fed sinapis pollen with a 21.8% protein content increased faster [33]. Moreover, an oilseed rape pollen and commercial pollen mix with a 22% protein content increased *B. terrestris* productivity and the size and longevity of male offspring [34] and reduced female mortality in the microcolony [35]. Pollen with a high ratio of protein and lipids can improve the collecting motivation of *B. impatiens* workers [36]. Nutrient-rich pollen can promote the health of bumblebees’ development and reproduction [29,37]. However, some research on *B. breviceps* feeding did not refer to the required pollen and nutrition [38,39]. Therefore, it is urgent to explore the nutritional requirements of *B. breviceps* in oviposition and the colony stage from the perspective of conventional pollen.

The research on the nutritional requirements of bumblebees mainly focuses on microcolonies of *B. terrestris* [32,40,41] or *B. impatiens* [36,42,43], which are composed of three-to-five workers per treatment group [34]. There are some advantages to using microcolonies over queenright colonies, such as the ease of creating and maintaining colonies, the possibility for larger sample sizes, and the easier standardization among microcolonies [44,45]; however, the nutritional conclusions based on laboratory-raised queenless microcolonies do not apply to queenright colonies of bumblebees in the wild, on account of the fact that a single queen collects pollen and nectar to supplement its nutrients after spring recovery. Therefore, it is more reliable to use a queen or queenright colony assessment than queenless microcolony assessments when trying to understand bumblebee queen nutritional requirements [35,46,47].

The key to artificially breeding the native bumblebee species *B. breviceps* is to find the nutrients it needs. Therefore, in this study, three types of dried pollen, including camellia pollen, oilseed rape pollen, and apricot pollen, were selected; these pollens are widely distributed in southern China and easily available. Then, these pollens, and their equivalent mixtures, were fed to *B. breviceps* queens to assess their performance, in order to provide a theoretical basis for the nutritional requirements of local bumblebees in reproduction and colony development.

## 2. Materials and Methods

### 2.1. Queen Collection, Rearing and Colony Management

The queens of *B. breviceps* in the wild were collected in the middle of the spring of 2021, in Guizhou Province, China. The queens and colonies were reared until virgin queens emerged. When the virgin queens matured, the queen mated with a drone from a different colony; method reference [32]. The queens and colonies were fed under the same conditions to maintain their similarity, using the method in [48], in the climate room of the laboratory of the College of Animal Science, Guizhou University. Successful mating queens of *B. breviceps* with a similar size, used for the colony foundation, were hibernated for 60 days at 4.5 ± 0.5 °C and 70% ± 5% relative humidity [48]. Sixty-three of the recovered queens were placed into a small plastic nest box (19 cm × 12.5 cm × 7.5 cm), where they were randomly assigned to one of the pollen treatments immediately and fed a sugar solution at a concentration of 50% as the diet (N = 9 queens per treatment) until the first worker emerged. Then, the queenright microcolonies were moved to a big plastic box (20 cm × 20 cm × 20 cm) and persistently fed until the tenth worker emerged. All queens and colonies were raised in an artificial climate chamber at 27 ± 1 °C and 70% ± 5% relative humidity, with an 8:16 weak-light cycle. Two types of boxes having a lid with a small hole were used to provide the pollen and sugar solution, allowing the bumblebees to feed freely. Sufficient equivalent pollen mass (at least 1 g) as treatment and 50% sugar solution were fed and replaced every two days. The feeding method followed that proposed by Jean Noël Tasei and Pierre Aupinel [41].

### 2.2. Pollen Selection and Treatments

Three pre-selected commercially sourced dried pollens, all collected from honeybee colonies with pollen traps, were purchased at one time from beekeepers. The three pollens were camellia pollen, oilseed rape pollen, and apricot pollen, which were obtained as camellia pollen mix, oilseed rape pollen mix, and apricot pollen mix with high proportions of a major pollen species and lower other diverse pollen species according to palynological analysis. We obtained seven pollen treatments as the diets, based on the three single pollen mixes and mixtures of two or three single pollen mixes in equal proportion. The seven experimental treatments were as follows: C: camellia pollen mix; O: oilseed rape pollen mix; A: apricot pollen mix; C–O: 50%:50% mixture of camellia pollen mix and oilseed rape pollen mix; C–A: 50%:50% mixture of camellia pollen mix and apricot pollen mix; O–A: 50%:50% mixture of oilseed rape pollen mix and apricot pollen mix; C–O–A: 1/3:1/3:1/3 mixture of the above three single pollen mixes. Each pollen treatment was well mixed again before being broken down, and then homogenized and stored in a freezer at −20 °C, in preparation for palynological analysis and feeding treatment. One of seven pollen treatments was offered to the queens and colonies (N = 9 queens per treatment).

### 2.3. Palynological Analysis

According to Smyth [49], a sample (0.5 g) of each pollen treatment was prepared for palynological identification and composition analysis. The sample of each pollen was placed in a drying oven for five hours at 40 °C, then moved and adhered to the microscope stage using conductive silver glue and sprayed gold for 3 min. A total of 150 grains of each pollen treatment were selected at random and identified to at least the genus level, and percentage compositions were counted using a scanning electron microscope with the acceleration voltage set to 5–15 kV. The morphology of the pollen was identified according to [50,51,52]. Pollen grains that could not be identified were recorded as “others”.

### 2.4. Determination of Pollen Nutritional Composition

Three subsamples (5–10 g) of each pollen mix treatment were tested in terms of nutritional composition, including crude protein content, lipid content, carbohydrate content, and amino acid content. The Bradford assay was used to calculate the protein concentration, and a modified assay from Van Handel and Day [53] was used to calculate carbohydrate and lipid concentrations. Amino acids were determined according to [32]. This determination method reflects the total amino acids (protein-incorporated and free in solution), excluding tryptophan and cysteine/cystine, which are usually lost during acid hydrolysis, and creatine and creatinine, which cannot be analyzed using this method. The amino acid content is presented as three groups, including essential amino acids (EAA), which must be obtained from the diet, non-essential amino acids (NEAA), which can be supplemented by the diet [54], and total amino acids (TAA), which is the sum of EAA and NEAA.

### 2.5. Observation of Queenright Colony Foundation

During the rearing period, the performance and state of the queens and queenright colonies were observed and recorded at a fixed time every day, including behaviors before egg hatching (nest building and egg laying), and the development of larvae, pupae, and workers. Thereinto, nine paracmeters were used to estimate colony performance for the treatment diets: (1) initial nesting time (for each queen, the time from the beginning of feeding on the treatment diet to the observation of secretory wax); (2) initial egg-laying time (for each queen, the time from the beginning of feeding on the treatment diet to the first observation of egg-laying activity); (3) number of eggs laid (the number of eggs laid by queens in the first batch); (4) the duration of larval development (the developmental duration of larvae in the first batch); (5) number of larvae ejected (the number of larvae ejected until the first worker emerged); (6) duration of pupal development (the developmental duration of pupae in the first batch); (7) time of first worker emergence (for each queen, the time from the beginning of feeding on the treatment diet to the emergence of the first worker); (8) the average weight of workers in the first batch; (9) the time it took the colony to reach ten workers (the interval from the initiation of feeding to the colony reaching ten workers). The terminology of other behaviors follows [27,32].

### 2.6. Statistics and Analysis

One-way ANOVA in SPSS 26.0 software was used to analyze the content of major nutrients in the pollen and the nine parameters of colony performance. Tukey post hoc tests were used to confirm where significant differences occurred between treatments. The results are expressed as the mean ± SE. The generalized linear model (GLM) method was used to analyze the data among parameters in colony performance and pollen nutrients. GLM selection was based on the Akaike information criterion (AIC). GraphPad Prism 9.4 was used for illustration. Values of *p* less than 0.05 and 0.01 indicate a significant difference and an extremely significant difference, respectively. Individual missing data were replaced with the group average. Data that did not conform to the normal distribution were converted with log or sqrt transformations.

## 3. Results

### 3.1. Palynological Analysis

Eight species of pollen among the seven treatments were identified using a scanning electron microscope. The camellia pollen mix mainly contained five pollen species, with 91.16% of the grains being *Camellia* sp. and the remainder dominated by *Brassica* sp., *Armeniaca* sp. *Lactuca sativa* and *Acer* sp. (Table 1). The oilseed rape pollen mix also mainly contained five pollen species, with 75.16% of the grains being *Brassica* sp. and 10.16% being *Armeniaca* sp.; the remaining grains were *Seriphidium* sp., *Cirsium japonicum* and *Acer* sp. (Table 1). The apricot pollen mix mainly contained three species of pollen, with 66.49% of the grains being *Armeniaca* sp. and 31.48% being *Brassica* sp.; the remaining grains were *Seriphidium* sp. The camellia–oilseed rape pollen mix mainly contained five species of pollen, including 47.13% *Brassica* sp. and 40.20% *Camellia* sp. (Table 1). The camellia–apricot pollen mix and oilseed rape–apricot pollen mix both principally contained three species of pollen, of which 44.36%, 34.25%, and 19.29% of the grains were *Camellia* sp., *Armeniaca* sp., and *Brassica* sp., respectively, in the camellia–apricot pollen mix, and 52.47%, 41.39% and 4.91% of the grains were *Brassica* sp., *Armeniaca* sp., and *Seriphidium* sp., respectively, in the oilseed rape–apricot pollen mix (Table 1). The camellia–oilseed rape–apricot pollen mix was the most abundant in pollen species, with 38.54% *Brassica* sp., 24.47% *Camellia* sp. and 22.10% *Armeniaca* sp.; the remaining grains were *Salix* sp. and *Seriphidium* sp. (Table 1).

### 3.2. Determination of Pollen Nutritional Composition

The crude protein content of the treatments ranged from 23.93% to 26.18%. There was a significant difference between the crude protein contents of the treatments (*F* = 8.96, df = 5, *p* = 0.0004; Table 2). The crude protein content was the highest in the oilseed rape pollen mix, where significant differences from the apricot pollen mix, camellia–oilseed rape pollen mix, camellia–apricot pollen mix, and oilseed rape–apricot pollen mix were found (*p* < 0.01; Table 2). The camellia–apricot pollen mix had the lowest crude protein content, where significant differences from the oilseed rape pollen mix and camellia–oilseed rape–apricot pollen mix were found (*p* < 0.05; Table 2). There was no significant difference between the camellia pollen mix, apricot pollen mix, camellia–oilseed rape pollen mix, camellia–apricot pollen mix, and oilseed rape–apricot pollen mix (*p* > 0.05; Table 2).

The lipid content ranged from 2.20% to 5.58%. The crude lipid content in the oilseed rape pollen mix was significantly higher than that of the other treatments (*F* = 315.1, df = 6, *p* < 0.05; Table 2), followed by the camellia–oilseed rape–apricot pollen mix and apricot pollen mix. The crude fat content in the oilseed rape–apricot pollen mix was the lowest, where significant differences from the oilseed rape pollen mix, apricot pollen mix, camellia–apricot pollen mix, and camellia–oilseed rape–apricot pollen mix were found (*p* < 0.05; Table 2). There was no significant difference between the camellia pollen mix, camellia–oilseed rape pollen mix, and oilseed rape–apricot pollen mix (*p* > 0.05; Table 2).

The carbohydrate content ranged from 25.99% to 47.22%. The carbohydrate content in the oilseed rape–apricot pollen mix was the highest, where significant differences from the camellia pollen mix, camellia–oilseed rape pollen mix, and camellia–apricot pollen mix were found (*p* < 0.05; Table 2); however, there was no significant difference from the other pollen treatments (*p* > 0.05; Table 2). The camellia pollen mix had the lowest carbohydrate content, which was significantly different from that of the other treatments (*F* = 85.86, df = 6, *p* < 0.01; Table 2).

The crude protein–lipid ratio (P: L) in all pollens ranged from 4.69 to 11.35 (Table 2). The carbohydrate–lipid ratio (C: L) ranged from 8.32 to 16.43 (Table 2). The carbohydrate–crude protein ratio (C: P) ranged from 1.04 to 1.89 (Table 2).

**Figure 1:**
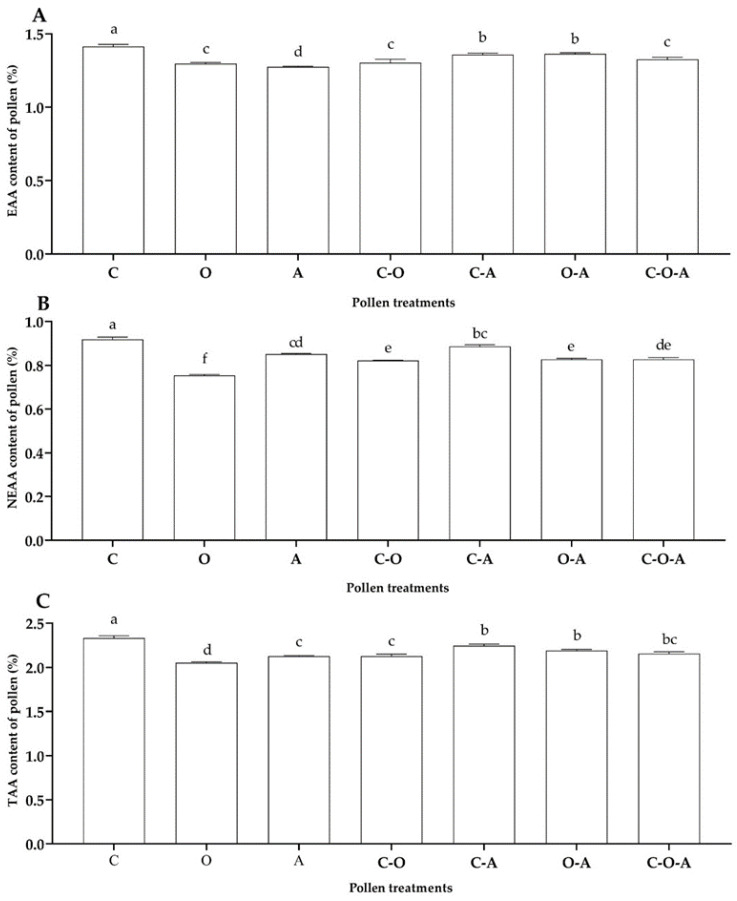
Contents of amino acids in the pollen treatments: (**A**) essential amino acids (EAA); (**B**) non-essential amino acids (NEAA); (**C**) total amino acids (TAA). C: camellia pollen mix; O: oilseed rape pollen mix; A: apricot pollen mix; C–O: camellia–oilseed rape pollen mix; C–A: camellia–apricot pollen mix; O–A: oilseed rape–apricot pollen mix; C–O–A: camellia–oilseed rape–apricot pollen mix. The data having same letter represents with no differences among treatments (*p* > 0.05), and the data having different letter represents with have significant differences among treatments (*p* < 0.05).

The essential amino acid (EAA) content ranged from 1.27% to 1.41%. The camellia pollen mix had a significantly higher EEA content than the other treatments (*F* = 98.98, df = 6, *p* < 0.01; Figure 1A), while the apricot pollen mix had the lowest, which was significantly lower than that of the other treatments (*p* < 0.01; Figure 1A). There was no significant difference between the oilseed rape–apricot pollen mix and camellia–apricot pollen mix; the same result was found between the oilseed rape pollen mix, camellia–oilseed rape pollen mix, and camellia–oilseed rape–apricot pollen mix (*p* > 0.05; Figure 1A).

The content of non-essential amino acids (NEAA) ranged from 0.75% to 0.92%. There was a significant difference in the NEAA content between the treatments (*F* = 56.73, df = 6, *p* < 0.01; Figure 1B). The NEAA content of the camellia pollen mix was significantly higher than that of the other treatments (*p* < 0.01; Figure 1B), followed by the camellia–apricot pollen mix and apricot pollen mix. There was no difference between the oilseed rape–apricot pollen mix, camellia–oilseed rape pollen mix, and camellia–oilseed rape–apricot pollen mix (*p* > 0.05; Figure 1B). The oilseed rape pollen mix had the lowest NEAA content, showing a significant difference from the other treatments (*p* < 0.05; Figure 1B).

There were significant differences in the total amino acid (TAA) contents among the seven treatments (*F* = 78.28, df = 6, *p* < 0.01; Figure 1C), ranging from 2.05% to 2.33%. The TAA content of the camellia pollen mix was significantly higher than that of the other treatments (*p* < 0.01; Figure 1C), while that of the oilseed rape pollen mix was the lowest, showing significant differences from the other treatments (*p* < 0.05; Figure 1C). There was no significant difference between the camellia–apricot pollen mix, oilseed rape–apricot pollen mix, and camellia–oilseed rape–apricot pollen mix (*p* > 0.05; Figure 1C); the same results were found between the apricot pollen mix, camellia–oilseed rape pollen mix and camellia–oilseed rape–apricot pollen mix (Figure 1C).

### 3.3. Observation of Queenright Colony Development

#### 3.3.1. Performance and Reproductive Time

**Figure 2:**
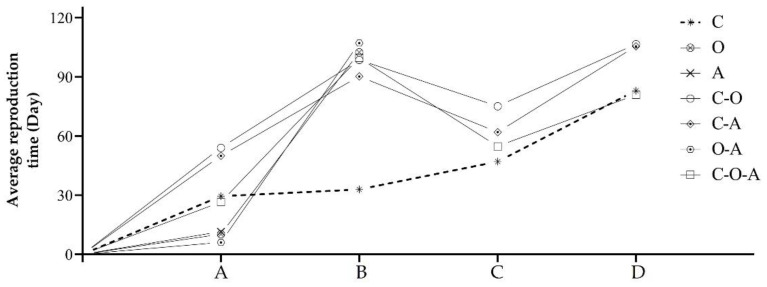
Reproductive time in the behavior of each pollen treatment (the deaths of queens were considered): (A) initial nesting; (B) initial egg laying; (C) first worker emergence; (D) colony reaching ten workers.

**Figure 3:**
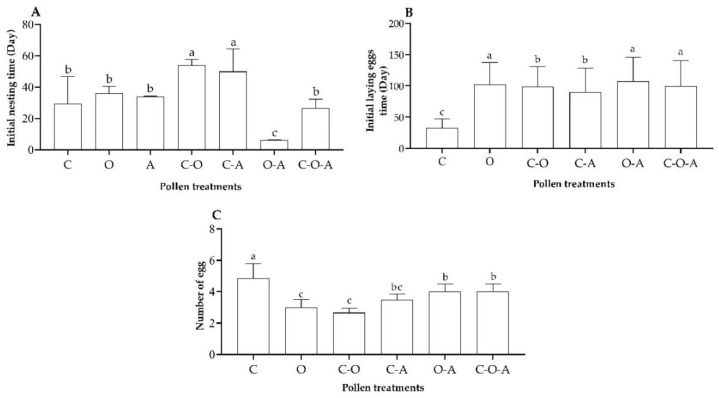
Parameters for the initial nesting and egg laying and larval ejection behaviors of each pollen treatment: (**A**) initial nesting time; (**B**) initial egg-laying time; (**C**) number of eggs. C: camellia pollen mix; O: oilseed rape pollen mix; A: apricot pollen mix; C–O: camellia–oilseed rape pollen mix; C–A: camellia–apricot pollen mix; O–A: oilseed rape–apricot pollen mix; C–O–A: camellia–oilseed rape–apricot pollen mix. The data having same letter represents with no differences among treatments (*p* > 0.05), and the data having different letter represents with have significant differences among treatments (*p* < 0.05).

The initial nesting behavior of all *B. breviceps* queens feeding on the pollen treatments was observed, ranging from 6 to 54 days, finding the shortest time in the oilseed rape–apricot pollen mix (Figure 2 and Figure 3A). Queens feeding on the apricot pollen mix treatment did not lay an egg until all queens died, while queens feeding on the other treatments all laid the first batch of eggs, with the initial egg-laying time ranging from 32 to 107 days, finding the shortest time in the camellia–oilseed rape pollen mix (Figure 2 and Figure 3B). In the development of larvae and pupae, larva ejection behavior occurred in six pollen mix treatments, of which all larvae were ejected in the oilseed rape–apricot pollen mix and oilseed rape pollen mix. The queens in the oilseed rape–apricot pollen mix and oilseed rape pollen mix both died quickly, while those in the remaining four treatments continued to develop (Figure 2). The first batch of worker emergence occurred in these four treatments, namely, the camellia pollen mix, camellia–oilseed rape pollen mix, camellia–apricot pollen mix, and camellia–oilseed rape–apricot pollen mix, of which the time of the first worker’s emergence in the camellia pollen mix was the shortest (Figure 2). It is important to note that partial queens were in three of the treatments. i.e., the camellia–oilseed rape pollen mix, camellia–apricot pollen mix, and camellia–oilseed rape–apricot pollen mix, had later initial nesting times, a higher death rate after laying eggs and lower colony foundation rates, which the mortality rate is 44.4%, 66.7%, and 77.8%, respectively, leading to the mean time of first worker emergence being lower than the initial egg-laying time (Figure 2). However, all workers of the above four treatments also reached a colony of ten workers, of which the time to reach a colony of ten workers for the camellia pollen mix and camellia–oilseed rape–apricot pollen mix was shorter than that of the other two treatments (Figure 2), while camellia pollen mix had a lower death rate (11.1%) and a higher colony foundation rate.

#### 3.3.2. Nest Building and Egg Laying

The initial nesting time of the experiment differed significantly among the pollen treatments (*F* = 27.82, df = 6, *p* < 0.01; Figure 3A). Tukey post hoc analyses confirmed that the initial nesting time in the oilseed rape–apricot pollen mix treatment was significantly shorter than that of the other treatments, and the times for the camellia–oilseed rape pollen mix and camellia–apricot pollen mix were both significantly longer than those of the other treatments (Figure 3A). There was no significant difference between the camellia pollen mix, oilseed rape pollen mix, apricot pollen mix, and camellia–oilseed rape–apricot pollen mix; the same result was found between the camellia–oilseed rape pollen mix and camellia–apricot pollen mix (*p* > 0.05; Figure 3A).

Unlike the queens fed the apricot pollen mix, which did not lay an egg until death, queens from the other six treatments laid the first batch of eggs. The initial egg-laying time in the camellia pollen mix was shorter than that of the other treatments (*F* = 5.885, df = 5, *p* < 0.05; Figure 3B), while the times for the oilseed rape pollen mix and oilseed rape–apricot pollen mix were longer than those of the other treatments (*p* < 0.05; Figure 3B), but there was no difference between these two groups (*p* > 0.05). There was no significant difference between the camellia–oilseed rape pollen mix and camellia–apricot pollen mix (*p* > 0.05; Figure 3B).

The number of eggs laid in the first batch of the experiment differed significantly among the pollen treatments. The number of eggs laid in the camellia pollen mix treatment was the highest, showing a significant difference from the other treatments (*F* = 18.49, df = 5, *p* < 0.05; Figure 3C). The number of eggs laid in the oilseed rape pollen mix and camellia–oilseed rape pollen mix treatments was significantly lower than that of the other treatments, except for the camellia–apricot pollen mix (*p* < 0.05; Figure 3C). There were no significant differences between the camellia–apricot pollen mix, oilseed rape–apricot pollen mix, and camellia–oilseed rape–apricot pollen mix (*p* > 0.05; Figure 3C).

#### 3.3.3. Development of Larvae and Pupae

**Figure 4:**
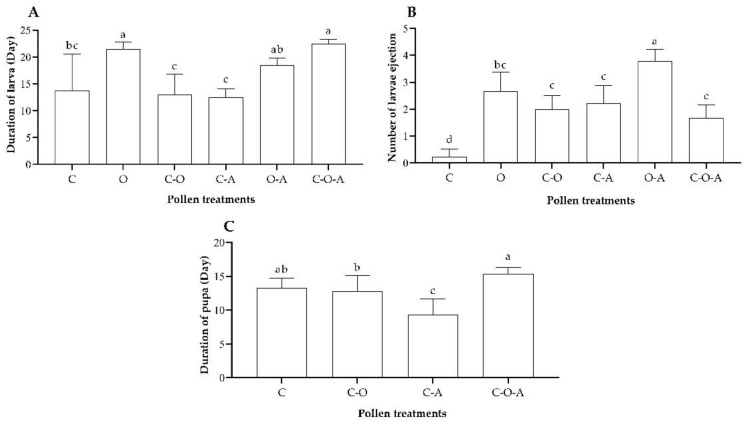
Parameters for the development of larvae and pupae of each pollen treatment: (**A**) duration of larval development; (**B**) number of larvae ejected; (**C**) duration of pupal development. C: camellia pollen mix; O: oilseed rape pollen mix; C–O: camellia–oilseed rape pollen mix; C–A: camellia–apricot pollen mix; O–A: oilseed rape–apricot pollen mix; C–O–A: camellia–oilseed rape–apricot pollen mix. The data having same letter represents with no differences among treatments (*p* > 0.05), and the data having different letter represents with have significant differences among treatments (*p* < 0.05).

The larval development duration ranged from 12 to 22 days. There was a significant difference in the larval development duration of the treatments (*F* = 15.76, df = 5, *p* < 0.0001; Figure 4A). The durations for the oilseed rape pollen mix, oilseed rape–apricot pollen mix, and camellia–oilseed rape–apricot pollen mix were longer than those of the other treatments (*p* < 0.05; Figure 4A), but there was no significant difference between them (*p* > 0.05; Figure 4A). The durations for the camellia–oilseed rape pollen mix and camellia–apricot pollen mix were shorter, showing significant differences from those of the other treatments (*p* < 0.05; Figure 4A), except the camellia–oilseed rape pollen mix. There was a significant difference in the number of larvae ejected (*F* = 42.83, df = 5, *p* < 0.01; Figure 4B), with the highest number for the oilseed rape–apricot pollen mix, where all larvae were ejected, showing a significant difference from the other treatments, followed by the oilseed rape pollen mix, where all larvae were ejected. The camellia pollen mix had the lowest number of ejected larvae, showing significant differences from the other treatments (*p* < 0.05; Figure 4B). There were no significant differences between the camellia–oilseed rape pollen mix, camellia–apricot pollen mix, and camellia–oilseed rape–apricot pollen mix (*p* > 0.05; Figure 4B).

As the queens of two treatments, namely, the oilseed rape pollen mix and oilseed rape–apricot pollen mix, all died in the larva stage, there were only four treatments in the pupa stage. The duration of pupal development ranged from 9 to 13 days in the camellia pollen mix, camellia–oilseed rape pollen mix, camellia–apricot pollen mix, and camellia–oilseed rape–apricot pollen mix (Figure 4C). The duration in the camellia–apricot pollen mix treatment was significantly shorter than that of the other treatments (*F* = 16.02, df = 3, *p* < 0.0001; Figure 4C). The duration in the camellia–oilseed rape–apricot pollen mix treatment was the longest, being significantly longer than that of the camellia–oilseed rape pollen mix and camellia–apricot pollen mix (*p < 0.05*; Figure 4C). There was no significant difference between the camellia pollen mix, camellia–oilseed rape pollen mix, and camellia–oilseed rape–apricot pollen mix (*p* > 0.05; Figure 4C).

#### 3.3.4. Development of Workers in Colony Foundation Stage

**Figure 5:**
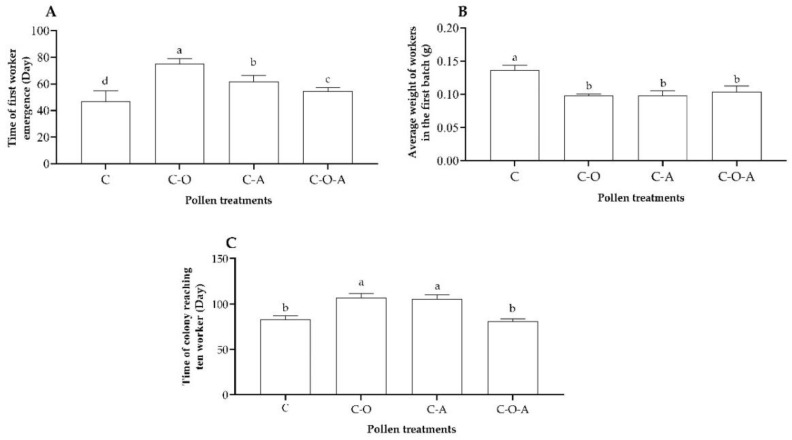
Parameters for the development of workers in each pollen treatment: (**A**) time of first worker emergence; (**B**) average weight of workers in the first batch; (**C**) time the colony took to reach ten workers. C: camellia pollen mix; C–O: camellia–oilseed rape pollen mix; C–A: camellia–apricot pollen mix; C–O–A: camellia–oilseed rape–apricot pollen mix. The data having same letter represents with no differences among treatments (*p* > 0.05), and the data having different letter represents with have significant differences among treatments (*p* < 0.05).

The time of first worker emergence differed significantly among four of the pollen treatments (*F* = 49.28, df = 3, *p* < 0.01; Figure 5A). For the camellia pollen mix, the time of first worker emergence was significantly shorter than that of the other three treatments (*p* < 0.05; Figure 5A). The time of first worker emergence was significantly longer for the camellia–oilseed rape pollen mix (*p* < 0.05; Figure 5A) than for the other three treatments.

The weight of workers in the first batch differed significantly among four of the pollen treatments (*F* = 66.55, df = 3, *p* < 0.01; Figure 5B). The weight in the camellia pollen mix was heavier than in the camellia–oilseed rape pollen mix, camellia–apricot pollen mix, and camellia–oilseed rape–apricot pollen mix (*p* < 0.01; Figure 5B), while there was no significant difference among these three treatments (*p* > 0.05; Figure 5B).

There was a significant difference in the time the colony took to reach ten workers (*F* = 110.9, df = 3, *p* < 0.0001; Figure 5C). The time in the camellia pollen mix treatment was significantly shorter than that in the camellia–oilseed rape pollen mix and camellia–apricot pollen mix treatments (*p* < 0.05), but with no difference from the camellia–oilseed rape–apricot pollen mix (*p* > 0.05; Figure 5C). The times in the camellia–oilseed rape pollen mix and camellia–apricot pollen mix treatments were longer, but there was no significant difference (*p* > 0.05; Figure 5C).

### 3.4. Analysis of the Relationship between Pollen Nutrition and Reproduction Performance

The relationship between reproduction performance and pollen nutrition was analyzed based on datasets of the major nutrients and their proportions and nine parameters in the seven pollen treatments. Pollen carbohydrate content significantly impacted the initial nesting time (*χ*^2^ = 6.323, *p* = 0.012). Pollen essential amino acid content, as a predictive variable, better predicted the initial egg-laying time (*χ^2^ =* 14.506, *p* < 0.0001) and the number of eggs laid (*χ^2^ =* 29.494, *p* < 0.0001). Lipid content better predicted the development duration of larvae (*χ^2^ =* 16.394, *p* < 0.0001) and pupae (*χ^2^ =* 4.826, *p = 0*.028). Pollen essential amino acid content, as a key variable, better predicted the time of first worker emergence (χ^2^ = 7.861, *p =* 0.005) and the average weight of workers in the first batch (*χ^2^ =* 12.221, *p* < 0.0001). Moreover, pollen crude protein content significantly impacted the time the colony took to reach ten workers (*χ^2^ =* 7.262, *p =* 0.007). The effects of total amino acid and non-essential amino acid contents and protein–lipid, carbohydrate–lipid, and carbohydrate–protein ratios on reproductive performance in each treatment were not significant (*p* > 0.05).

## 4. Discussion

Pollen contains protein, lipids, carbohydrates, sterols, vitamins, and minerals [55], which are all nutrients needed by honeybees [56], but the nutrients in pollen from different plant species vary, containing 2.5–61% of crude protein and 2–20% of crude fat [28]. Bumblebees need to obtain diverse nutrients from pollen to meet their developmental demands [57]. The inequality of nutrients in different pollen sources implies that the performance of bumblebees might be affected. Therefore, in this study, different nutritive pollen mixes were used to evaluate the performance of *B. breviceps* queens and to explore the nutritional needs of their growth and reproduction.

According to the performance of the queens and colonies feeding on different pollens, we found that not all pollen treatments can meet the growth and development needs of *B. breviceps* queens and colonies. In our study, the queens feeding on the apricot pollen mix did not lay eggs, while the queens feeding on the oilseed rape pollen mix and oilseed rape–apricot pollen mix did lay eggs; nevertheless, all larvae were ejected until the queens died. Fortunately, the queens feeding on the camellia pollen mix with a higher amino acid content and higher protein content had a significant advantage in the initial egg-laying time, the number of eggs laid, duration of larval development, the number of larvae ejected, time of first worker emergence, the average weight of workers in the first batch and the time the colony took to reach ten workers. The queens feeding on the oilseed rape–apricot pollen mix had advantages in the initial nesting time, and the queens feeding on the camellia–apricot pollen mix had a distinct advantage in the duration of larval and pupal development.

The protein content is generally used to estimate the pollen quality and nutritive value for bumblebees; high-protein pollen seems to reduce the mortality of larvae and females and the larval ejection rate, and increase the offspring number and larval weight [33]. Oilseed rape pollen with 22% protein content, and sunflower pollen with 13% protein content were fed to a *B. terrestris* microcolony. The workers with higher protein had higher productivity, and the drones were heavier [34]. In another study, a queenright *B. terrestris* colony was fed pollens containing 22%, 18%, and 14% crude protein; the results showed that the pollen group with 22% protein content had lower larval mortality and heavier weighted workers than the pollen group with 14% protein content [35]. In our study, combined with the reproduction performance and pollen nutrition, the results show that the higher crude protein and essential amino acid contents in the camellia pollen mix can contribute to the strong performance of *B. breviceps* queens and colonies. On the contrary, the lower crude protein and essential amino acid contents in the apricot pollen mix cause *B. breviceps* queens not to lay eggs, as well as their death. However, the relationship based on the major nutrients and their proportions and the nine parameters in the pollen shows that pollen with a higher essential amino acid content has advantages in the initial egg-laying time, the number of eggs laid, time of first worker emergence, and the average weight of workers in the first batch, while a higher protein content can only accelerate the colony development. Although high-protein pollens generally contribute more to colony growth and development than low-protein pollens [58,59], the nutritional quality of high-protein pollen is reduced if there are inadequate amounts of the essential amino acids required for growth [58,60]. Therefore, the composition and content of amino acids can be used to more accurately evaluate the nutritional value of pollen than the content of protein [61,62].

Some studies have shown that the content of amino acids in pollen is the main driving factor for the normal development of bumblebees [21,31], especially the content of essential amino acids [32,63]. Pollens with a high proportion of essential amino acids are considered to have higher nutritional value than those with a low proportion of essential amino acids [55]. According to one study, bumblebees in the field generally prefer to collect pollen with a significantly higher essential amino acid content [63]. A microcolony of *B. terrestris* had the worst performance in the initial egg-laying time when feeding on rape pollen with a lower essential amino acid content; in contrast, the performance of the microcolony was better in the initial egg-laying time and the number of larvae ejected when feeding on camellia pollen with a higher essential amino acid content [32]. Previously, three types of pollen, namely, *Actinidia deliciosa*, *Cistus* sp., and *Salix* sp., with different contents of essential amino acids were used to feed a *B. terrestris* microcolony. The results showed that the pollen with a higher content of essential amino acids had higher egg production, the lowest number of ejected larvae, and the heaviest pupae [31]. The reasons for the influence of essential amino acids on the initial egg-laying time and the number of larvae ejected may be that a higher essential amino acid content stimulates the female ovaries to accelerate development [64], promotes hormone synthesis, gene expression, and functional development of the cell membrane in larvae [65]. Thus, queens or workers accelerate egg laying and larval maturation [31]. This may be the reason why the camellia pollen mix with a higher content of essential amino acids in our experiment showed a better performance.

Similar to some research results, our results suggest that it took a shorter amount of time for the colony to reach ten workers under the camellia pollen mix and camellia–oilseed rape–apricot pollen mix, with higher protein contents. Wynants et al. reported that protein contents of 21.8%, 21.4%, 18.4%, and 11.9% were fed to a queenright *B. terrestris* colony, and they found that the number of offspring with a higher protein content increased faster [35]. This may be because when the first worker emerges successfully, it can continue to feed on pollen with high protein and essential amino acid contents, which allows the worker’s body size to develop faster, and helps the queen to brood, thus reducing the time required for the colony to reach ten workers.

The lipid content of pollen is also a factor affecting the development of bumblebees [42]. In our study, in general, the duration of larval and pupal development lengthened with the increase in the lipid content in the treatments, except for the camellia–apricot pollen mix. The queens feeding on the camellia–apricot pollen mix with a higher lipid content had a shorter duration of larval and pupal development. According to previous research, the essential sterol in pollen is the precursor of ecdysone, which is crucial to the development of honeybees’ larvae [66,67]. High lipid content in pollen seems to be detrimental to the survival and reproduction of bumblebees and the development of larvae [68], but its impact mechanism is not completely clear. Noteworthy, the average pollen P:L value was 14:1 for *B. terrestris* workers and 12:1 P:L for *B. impatiens* workers when fed in limited conditions [42]. Therefore, we speculate that multiple factors not considered, such as the proportion of nutrients and sterols, jointly led to the difference in the duration of larval and pupal development under the camellia–apricot pollen mix.

The effects of carbohydrates in pollen on bumblebees have been poorly studied. In one report of a queenright *B. breviceps* microcolony fed different sugar solutions, the pre-oviposition period of the bumblebees varied with the type of sugar syrup [38]. In our study, the queens feeding on the oilseed rape–apricot pollen mix with a higher carbohydrate content had advantages in the initial nesting time. Meanwhile, previous studies have shown that amino acids can affect the initial nest-building time [32]. This may be due to the combined effect of higher protein, NEAA, EAA contents, and lower lipid contents in the pollen, which meet their needs, thus increasing the demand for carbohydrates, but the specific reasons need to be further studied.

In addition, in our study, the performance of the queens and queenright colonies was worse than that found in other studies on *B. breviceps* and *B. terrestris*. More specifically, the mortality rate of the queens was higher compared with the female mortality of *B. terrestris* when raised with or without a queen [32,35]. Some studies deem that the high death rate is caused by pollen with a low protein or amino acid content [40,69] or the preservation time of pollen [70]. Therefore, we speculate that the death of queens may be caused by the pollen not being sufficient to meet their nutritional needs, artificial diapause unreasonably, degree of domestication, or some undetected toxic substances in the pollen. Additionally, the initial egg-laying time, ranging from 32 to 107 days, is distinctly longer than that found for *B. breviceps* and *B. terrestris* in previous research. For example, Liang et al. and Qin reported that the average egg-laying time of *B. breviceps* was 13.6 days and 8.9–15.2 days at different collection sites in queenright colonies, respectively [27,39], but they unpublished feeding pollen. Other studies have shown that *B. terrestris* queen’s average initial egg-laying time ranges from 11.3 to 12.5 days [33]. The time of the first worker’s emergence was also longer in our study. In pollen nutrition tests of *B. terrestris*, workers are often added to stimulate queen ovarian development to speed up egg production [33]. However, after hibernation in the wild environment, there are no workers to assist in egg laying; we simulated this real environment in the wild. In addition, compared with fresh pollen, dried pollen will significantly affect the time before egg laying [70]. Therefore, we speculate that the differences in performance were due to the rearing environment, such as temperature and humidity, as well as rearing techniques, species, etc.

To sum up, the content and proportion of major nutrients in different pollens, especially the contents of essential amino acids and protein, seem to lead to differences in the indicators of queenright *B. breviceps* colonies, such as the initial egg-laying time, the average weight of the first batch of workers, the time of first worker emergence and the time the colony takes to reach ten workers. However, it is noteworthy that our research is restricted to the main macroelement nutrients, while other nutrients are not considered, such as sterol concentration [61], fatty acids [71], taste [72], and digestibility [73]. Therefore, there is more comprehensive work to be conducted to understand the needs of *B. breviceps* in terms of growth and reproduction.

## 5. Conclusions

This study evaluated the feasibility of feeding queens *B. breviceps* different pollens from the perspective of some major nutrients. The results showed that the camellia pollen mix with a high essential amino acid content accelerated *B. breviceps* queens’ initial egg-laying time, increased the number of eggs and the average weight of workers in the first batch, and reduced the rate of larval ejection and the time of first worker emergence, while the camellia pollen and camellia–oilseed rape–apricot pollen mix with a high protein content helped the colony foundation of the workers. The results provide data for ascertaining the reproductive nutritional needs of local bumblebee species and, further, for protecting local bumblebee species.

## Figures and Tables

**Table 1:** The composition and percentage of the pollen treatments.

Pollen Treatments	*Brassica* sp. (%)	*Camellia* sp. (%)	*Armeniaca* sp. (%)	*Seriphidium* sp. (%)	*Cirsium* sp. (%)	*Lactuca* sp. (%)	*Acer* sp. (%)	*Salix* sp. (%)	Others (%)
C	3.32	91.16	1.32	-	-	1.05	1.05	-	2.11
O	75.16	-	10.16	4.15	0.71	-	-	9.10	0.71
A	31.48	-	66.49	1.35	-	-	-	-	0.67
C–O	47.13	40.20	7.44	3.21	-	1.01-	-	-	1.01
C–A	19.29	44.36	34.25	-	-	-	-	-	2.10
O–A	52.47	-	41.39	4.91	-	-	-	-	1.23
C–O–A	38.54	24.27	22.10	6.06	-	1.01	-	7.01	1.01

Notes. C: camellia pollen mix; O: oilseed rape pollen mix; A: apricot pollen mix; C–O: camellia–oilseed rape pollen mix; C–A: camellia–apricot pollen mix; O–A: oilseed rape–apricot pollen mix; C–O–A: camellia–oilseed rape–apricot pollen mix; “-” stands for “none”.

**Table 2:** Major nutrients and proportions of the pollen treatments.

Pollen Treatments	Crude Protein (%)	Lipid (%)	Carbohydrate (%)	P: L	C: L	C: P
C	25.00 ± 0.06 ^abc^	2.49 ± 0.19 ^e^	25.99 ± 4.29 ^d^	10.01	10.40	1.04
O	26.18 ± 2.03 ^a^	5.58 ± 1.61 ^a^	46.44 ± 11.97 ^a^	4.69	8.32	1.77
A	24.67 ± 1.07 ^bc^	4.48 ± 0.05 ^c^	46.24 ± 6.23 ^a^	5.51	10.33	1.87
C–O	24.76 ± 2.45 ^bc^	2.23 ± 0.02 ^e^	36.56 ± 4.77 ^b^	11.13	16.43	1.48
C–A	23.93 ± 3.71 ^c^	3.55 ± 0.24 ^d^	31.62 ± 5.95 ^c^	6.74	8.91	1.32
O–A	24.97 ± 1.09 ^bc^	2.20 ± 0.47 ^e^	47.22 ± 3.24 ^a^	11.35	21.46	1.89
C–O–A	25.70 ± 1.18 ^ab^	5.03 ± 0.05 ^b^	45.12 ± 11.25 ^a^	5.11	8.97	1.76

Notes. C: camellia pollen mix; O: oilseed rape pollen mix; A: apricot pollen mix; C–O: camellia–oilseed rape pollen mix; C–A: camellia–apricot pollen mix; O–A: oilseed rape–apricot pollen mix; C–O–A: camellia–oilseed rape–apricot pollen mix. The data having same letter represents with no differences among treatments (*p* > 0.05), and the data having different letter represents with have significant differences among treatments (*p* < 0.05).

## Data Availability

Original data at https://figshare.com/articles/dataset/Feeding_data/22109018.
